# Resveratrol suppresses malignant progression of oral squamous cell carcinoma cells by inducing the ZNF750/RAC1 signaling pathway

**DOI:** 10.1080/21655979.2021.1940616

**Published:** 2021-06-27

**Authors:** Yue Xiao, Yanjun Duan, Yongjie Wang, Xiaojia Yin

**Affiliations:** a Department of stomatology, Xiangyang No.1 People’s Hospital, Hubei University of Medicine, Xiangyang, 441000, Hubei Province, P.R.C; b Department of Stomatology, Xiangyang Central Hospital, Affiliated Hospital of Hubei University of Arts and Science, 441021, Xiangyang, Hubei, China

**Keywords:** Resveratrol, oral squamous cell carcinoma, ZNF750/RAC1 pathway, proliferation, apoptosis

## Abstract

This study examined whether activation of zinc finger protein 750/Ras-related C3 botulinum toxin substrate 1 (ZNF750/RAC1) signaling pathway may be involved in the ability of resveratrol to inhibit malignant progression of CAL-27 oral squamous cell carcinoma cells. CAL-27 cells were treated with resveratrol and transfected with plasmids expressing a ZNF750 mimic or ZNF750 inhibitor. Cell proliferation and apoptosis were assessed. Western blotting was used to examine the effects of resveratrol on levels of angiogenin, vascular endothelial growth factor (VEGF), prolyl hydroxylase 2 (PHD2), G protein signal-regulated protein 5 (RGS5), integrin A5 (ITGA5), integrin B1 (ITGB1), CD44, RAC1, and ZNF750. Quantitative PCR was used to examine the effects on mRNA levels of platelet-derived growth factor (PDGFB), tumor vascular marker CD105, and cell adhesion molecules ITGA5, ITGB1, and CD44. Resveratrol downregulated angiogenin, VEGF, RGS5, CD105, and the cell adhesion molecules ITGA5, ITGB1, and CD44 expressions to inhibit the vascular normalization, metastasis, adhesion, and migration of CAL-27 cells. Conversely, it upregulated ZNF750, PHD2, and PDGFB to suppress the malignant progression of CAL-27 cells. We further observed that these changes were associated with reduced proliferation, reduced colony formation, and increased apoptosis in cancer cells. ZNF750 silencing partly reversed these effects of resveratrol on the proliferation and apoptosis of CAL-27 cells. Additionally, RAC1 agonist also weakened these impacts of resveratrol on the growth of CAL-27 cells. The ability of resveratrol to suppress the progression of oral squamous cell carcinoma may involve activation of the ZNF750/RAC1 signaling pathway and modification of the tumor vascular microenvironment.

## Graphical abstract

**Figure:**
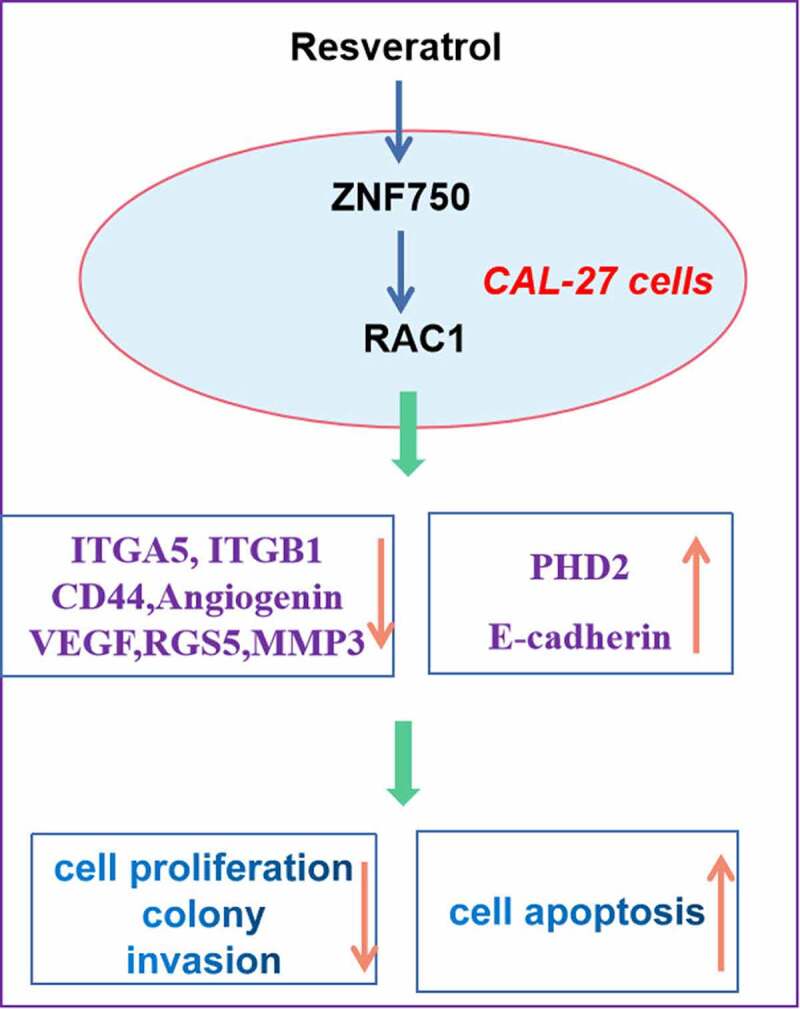

## Highlights

- Resveratrol inhibits the malignant progression of CAL-27 cells.
- Resveratrol induces ZNF750/RAC1 pathway.
- Resveratrol decreases CAL-27 cell growth via ZNF750/RAC1 axis.

## Introduction

Targeted molecular therapies inhibit oncogenic-signaling pathways necessary for progression of malignant cancers [1]; for example, monoclonal antibodies may bind key protein actors, and small-molecule kinase inhibitors may inhibit phosphorylation-mediated activation of key proteins. Various genetic variants have been associated with the development of human oral squamous cell carcinoma (OSCC) [2–4], raising the possibility that the corresponding proteins can be targeted to inhibit their involvement in oncogenic pathways.

Deletion or mutation of zinc finger protein 750 (ZNF750) has been linked to OSCC as well as to malignant phenotype in squamous epithelial cell cultures [5]. Indeed, ZNF750 appears to be a lineage-specific tumor suppressor gene in OSCC as well as a promising biomarker of poor outcomes in patients with the disease [6–8]. ZNF750 may suppress malignant progression of OSCC by regulating the tumor vascular microenvironment [9]. Additionally, Ras-related C3 botulinum toxin substrate 1 (RAC1) is a member of the Rho/Rac GTPase family by binding its promoter region, which can be found either in an active state, when complexed to GTP, and in an inactive state when bound to GDP [10–12]. When activated, RAC1 is involved in the cell migration, through a cytoskeleton rearrangement, and in cell survival [13–15]. Furthermore, ZNF750 represses the expression of RAC1 in breast cancer cell lines, by directly binding its promoter region [16].

The tumor vascular microenvironment contributes to tumorigenesis by providing oxygen, nutrients, and soluble growth factors. Hypoxia and limited nutrients in this microenvironment can trigger the production of angiogenic cytokines, such as VEGF, triggering vascular abnormalities, and cancer progression [9]. The tissue oxygen sensing PHD2 is an important mediator of vascular normalization and regulator of RGS5, which is responsible for vessel maturation. PDGFB is responsible for vessel maturation [17]. CD44, a transmembrane adhesion receptor for hyaluronan and growth factors, contributes to the migration and invasion by tumor cells [18]. Moreover, ITGB1 and ITGA5 mainly participant in cancer cell adhesion, cell migration, and invasion [19,20].

Given the ability of the natural phytoalexin resveratrol to reduce the risk of OSCC as well as other cancers [21], we wondered whether this compound may interact with the endogenous tumor-suppressing activity of ZNF750/RAC1 signaling pathway in OSCC cells. Therefore, the current study examined whether the inhibitory effects of resveratrol on OSCC malignant phenotype involve activation of ZNF750/RAC1 cascade.

Here, we supposed that resveratrol could decrease the growth of OSCC cells by inducing ZNF750/RAC1 signaling pathway. To begin to identify its anti-cancer mechanisms of action, this study examined the effects of resveratrol on the growth and invasion of OSCC cells, as well as the potential role of ZNF750/RAC1 in mediating those effects.

## Material and methods

### Reagents

Resveratrol, the RAC1 inhibitor (NSC23766), and RAC1 agonist (deacetylmycoepoxydiene, DAM) were purchased from Sigma (St. Louis, MO, USA). Antibodies against the following proteins were obtained from Abcam Signaling Technology (Cambridge, UK): angiogenin (#ab1198321), vascular endothelial growth factor (VEGF, #ab1231474), prolyl hydroxylase 2 (PHD2, #ab1769832), G protein signal-regulated protein 5 (RGS5, #1122176), integrin A5 (ITGA5, #1043208), integrin B1 (ITGB1, #1567432), CD44 (#1431347), RAC1 (#1475521) and ZNF750 (#1098453). The CCK-8 kit was purchased from Huamei (Wuhan, China). Annexin V-FITC and propidium iodide (PI) kits were purchased from BD (Franklin Lakes, NJ, USA). The BrdU ELISA kit was purchased from Usabio.cn. Trizol reagent was obtained from Thermo Fisher Scientific (Franklin Lakes, MO, USA).

### Cell culture and treatments

CAL-27 OSCC cells were provided by the Human Science Research Resources Bank and cultured in DMEM supplemented with 10% fetal bovine serum at 37℃ in an environment of saturated humidity and 5% CO_2_. Cells in the logarithmic growth phase were used in all experiments. Resveratrol was dissolved in DMSO and added to cells at final concentrations of 10, 20, or 40 μM. Control cells were given the same volume of DMSO vehicles. Cells were incubated with resveratrol for 48 h. Each experiment was performed in triplicate.

### Transfection with plasmids encoding ZNF750 mimic or ZNF750 inhibitor

To test the effects of ZNF750 overexpression and knockdown, CAL-27 cells were transiently transfected with empty vector (#20190112, Santa Cruz Biotechnology, AB, USA) or the same vector encoding the ZNF750 mimic (#20190123) or the ZNF750 inhibitor (#20190126). Cells were transfected using Lipofectamine 3000 (Invitrogen) based on the manufacturer’s instructions. ZNF750 overexpression and knockdown were confirmed by Western blot.

### Cell viability and proliferation

After CAL-27 cells had been treated with resveratrol (10, 20, or 40 μM) for 48 h, the culture medium was removed completely, and 100 μL of medium containing 10 μL of CCK-8 reagent was added to each well. Plates were incubated for 2 h, then optical density (OD) at 540 nm was measured using a microplate reader (Bio-Rad, Hercules, CA, USA). Relative cell viability (%) was calculated as OD_experiment/_OD_control_ × 100%.

Treated cells were lysed in RIPA lysis buffer (Beyotime Biotechnology, Shanghai, China) and analyzed by BrdU ELISA based on the manufacturer’s instructions. OD at 507 nm was measured using a microplate reader (Bio-Rad).

### Colony formation

After CAL-27 cells had been treated with resveratrol (10, 20, or 40 μM) for 6 days, numbers of cell colonies were counted under a reversed microscope. For each condition, the numbers of colonies in 10 fields of view were averaged.

### Apoptosis

After CAL-27 cells had been treated with resveratrol (10, 20, or 40 μM) for 48 h, cells were harvested and adjusted to a concentration of 1 × 10^6^ cells/mL. The cell suspension (0.5 mL) was stained with 1.25 μL Annexin V-FITC at room temperature for 15 min in the dark, then 10 μL PI was added. Cells were sorted by apoptosis stage using a BD flow cytometer.

## Cell invasion

Cell invasion assay was performed as describing in the previous study [22]. Briefly, cancer cells were plated in the upper chamber above the Matrigel at a density of 2 × 10^4^ cells/well, while the lower chamber contained DMEM medium with 10% FBS. After treatment with resveratrol (10, 20, and 40 μM) for 48 h, the membranes were recovered, and excess cells were removed from the upper membrane surface. The recovered membranes were then fixed with 4% paraformaldehyde at room temperature and stained with 1 μg/mL 4´,6-diamidino-2-phenylindole. Their underside was photographed, and the stained cells were counted under an inverted fluorescent microscope (BD, Franklin Lakes, NJ, USA).

### Levels of target mRNAs

After CAL-27 cells had been treated with resveratrol (10, 20, or 40 μM) for 48 h, cells were washed twice with phosphate-buffered saline (PBS) and harvested. Total RNA was extracted using Trizol reagent. RNA concentration and purity were assessed using the NanoDrop 2000 (Thermo Fisher). RNA (1 μg) was reverse-transcribed in a 20-μL reaction using a PrimeScript^@^ RT Kit. Then quantitative PCR was performed using the SYBR® Premix Ex Taq™ II kit (Takara, Dalian, China) on an ABI 7500 Sequence Detection System (Applied Biosystems, NJ, USA). Amplification parameters were 30 s pre-incubation at 95°C, followed by 40 cycles of 95°C for 5 s and 60°C for 34 s. The following primers were used: PDGFB forward, 5′-TCCCGAGGAGCTTTATGAGA-3′ PDGFB reverse, 5′-GGGTCATGTTCAGGTCCAAC-3′ CD105 forward, 5′-CCTCTACCTCAGCCCACACT-3′ CD105 reverse, 5′-TCTAACTGGAGCAGGAACTCG-3′ β-actin forward, 5′-CTACCTCATGAAGATCCTCACC-3′ and β-actin reverse, 5′-AGTTGAAGGTAGTTTCGTGGA T-3′. Fold changes in the two target genes were normalized to levels of β-actin using the 2^−ΔΔCT^ method. Samples were analyzed in duplicates and each experiment was performed three times.

### Levels of target proteins

After CAL-27 cells had been treated with resveratrol (10, 20, or 40 μM) for 48 h, cells were harvested and total protein was extracted using RIPA lysis buffer. Total protein concentration was estimated using the Bradford method (Thermo Fisher Scientific). Proteins (50 μg) were fractionated using 10% sodium dodecyl sulfate-polyacrylamide gel electrophoresis and transferred onto nitrocellulose membranes. Nonspecific binding sites were blocked with 5% skim milk for 1.5 h at room temperature on a shaking table. Then blots were incubated overnight at 4℃ with rabbit anti-mouse monoclonal antibodies (all diluted 1:1000) against the following proteins: angiogenin, VEGF, PHD2, RGS5, ITGA5, ITGB1, CD44, and ZNF750. Subsequently, blots were washed three times with PBS-Tween 20, and blots were incubated for 2 h at room temperature with horseradish peroxidase-conjugated goat anti-rabbit antibody (diluted 1:4000). Proteins were detected using a luminol reagent and peroxide solution (Millipore, Billerica, MA, USA). Densitometry of images was performed using Image J software.

### Statistical analysis

Data were reported as mean ± SD. Inter-group differences were assessed for significance using Welch’s *t* test (comparisons of two groups) or ANOVA (comparisons of three or more groups). A value of *P*< 0.05 was considered significant.

## Results

In our study, we supposed that resveratrol could suppress the proliferation of CAL-27 cells by inducing ZNF750/RAC1 signaling pathway. To confirm the anti-cancer effects of resveratrol, we first observed its effects on the growth of OSCC cells. Then, the potential roles of ZNF750/RAC1 signal transduction in OSCC cells were explored.

### Impact of resveratrol on proliferation and apoptosis

**Figure 1.**
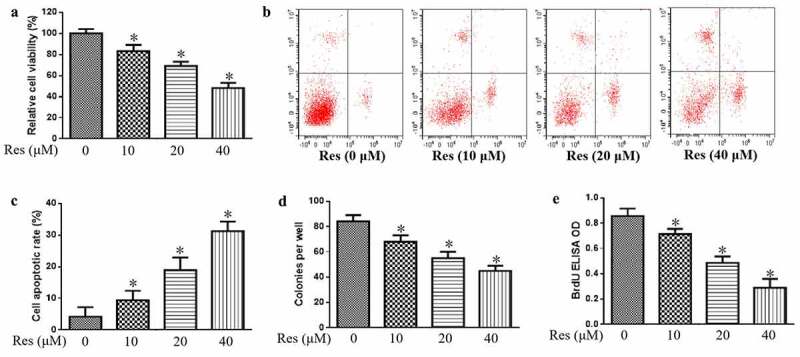
Effects of resveratrol (Res) on malignant phenotype of OSCC cells. Treated cells were assayed for (a) viability, (b) apoptosis, (c) colony-forming ability, and (d) proliferation based on BrdU incorporation. *P< 0.05 vs. Res 0 μM control

In addition, we also study the impact of resveratrol on the growth of CAL-27 cells. Our data indicate that resveratrol significantly decreased the viability of CAL-27 cells in a concentration-dependent manner based on the CCK-8 assay (Figure 1(a)), which coincided with significant upregulation of apoptosis based on flow cytometry (Figure 1(b)). Resveratrol also significantly decreased the number of colonies and BrdU-positive cells in a concentration-dependent manner (Figure 1(c-d)). These findings suggest that resveratrol could inhibit the proliferation and induce the apoptosis of cancer cells to decrease malignant progression of oral squamous cell carcinoma cells.

### Impact of resveratrol on cell invasion ability and the expressions of cell adhesion molecules ITGA5, ITGB1, and CD44

**Figure 2.**
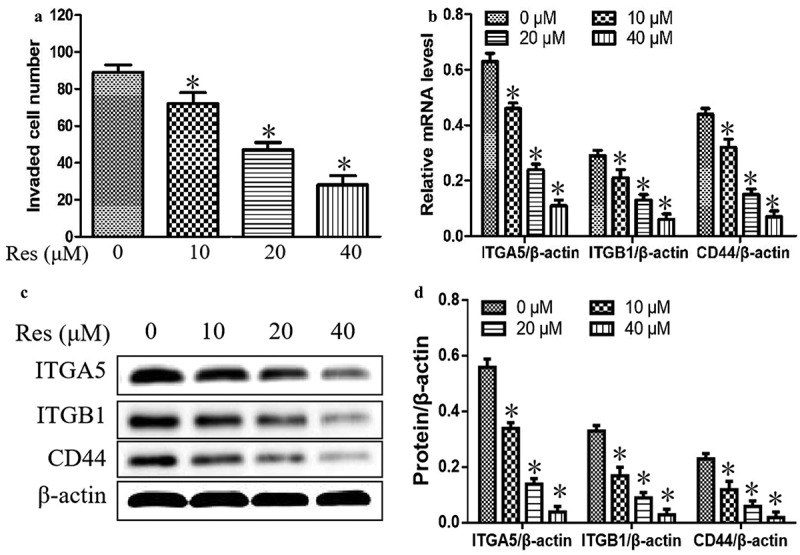
Effects of resveratrol (Res) on cell invasion and the expressions of cell adhesion and migration molecules ITGA5, ITGB1, and CD44. After treatment with 0, 10, 20, and 40 μM resveratrol for 48 h, (a) the number of invading CAL-27 cells was determined by the Matrigel invasion assay; (b) the relative expression levels of ITGA5, ITGB1, and CD44 mRNA were measured by qRT-PCR; the relative expression levels of ITGA5, ITGB1, and CD44 were determined using (c) Western blotting and (d) quantitation. *P< 0.05 vs. Res 0 μM control

We further explore the effect of resveratrol on the invasion and adhesion abilities of oral squamous cell carcinoma cells. As shown in Figure 2(a), cell invasion was significantly reduced with increasing resveratrol concentrations compared to cells incubated without resveratrol. Additionally, resveratrol significantly downregulated the mRNA and protein levels of ITGA5, ITGB1and CD44, which are involved in cell adhesion and cell migration, in a concentration-dependent manner (Figure 2(b-d)). Our results suggested that resveratrol could suppress the adhesion and migration in CAL-27 cells.

### Impact of resveratrol on expression of genes related to vascular normalization and metastasis

**Figure 3.**
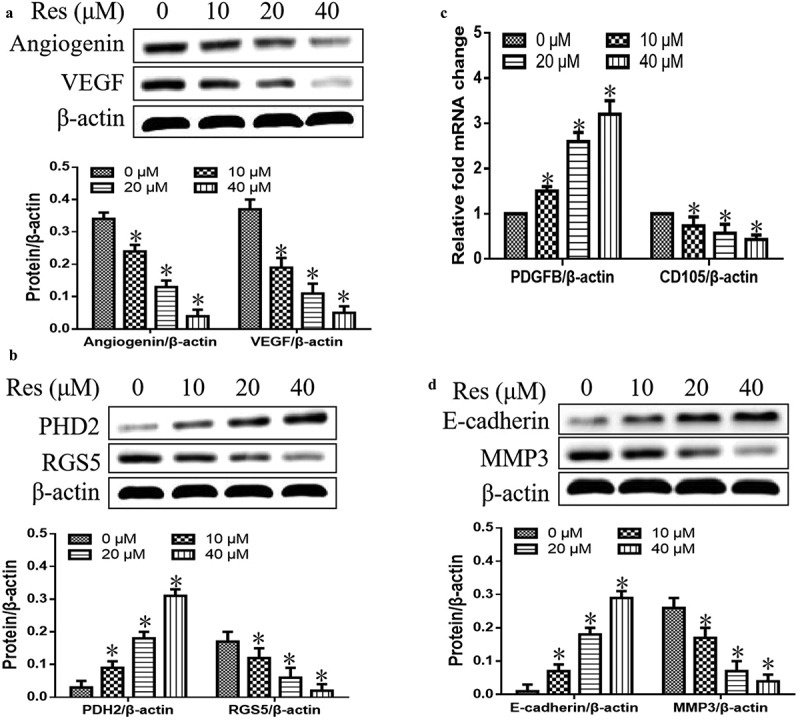
Effects of resveratrol (Res) on expression of genes associated with vascular normalization and metastasis: (a) angiogenin and VEGF, (b) PHD2 and RGS5, (c) PDGFB and CD105, and (d) E-cadherin and MMP3. *P< 0.05 vs. Res 0 μM control

We first observed the effects of resveratrol on the expression levels of proteins or genes associated with vascular normalization and metastasis. As shown in Figure 1, we found that resveratrol significantly inhibited expression of angiogenin and VEGF in a concentration-dependent manner (Figure 3(a)). It also downregulated RGS5, CD105, and MMP3, while upregulating PHD2, PDGFB and E-cadherin with a dose-dependent manner (Figure 3(b-d)). These findings showed that resveratrol could strongly suppress the vascular normalization and metastasis in oral squamous cell carcinoma cells.

### Impact of resveratrol on ZNF750/RAC1 signaling pathway

**Figure 4.**
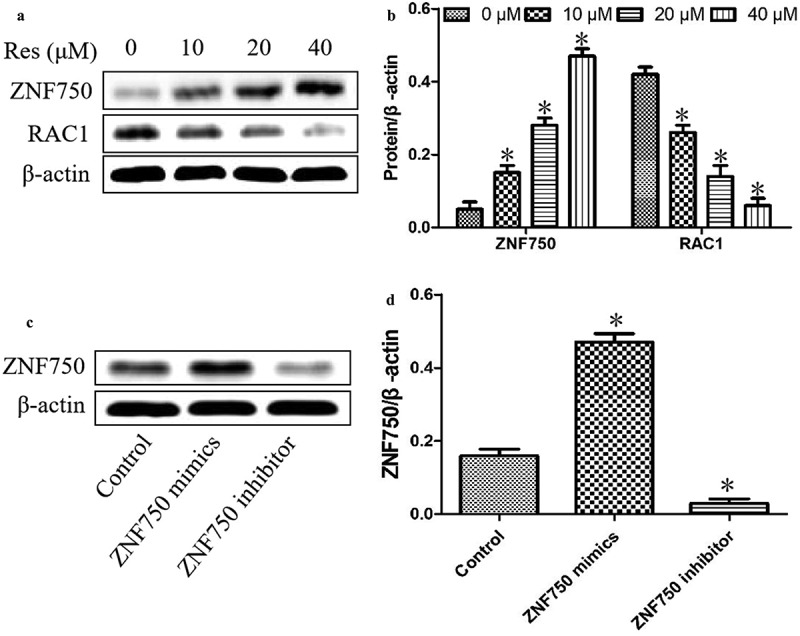
Effects of resveratrol (Res) on ZNF750 and RAC1 expressions. After treatment with 0, 10, 20, and 40 μM resveratrol for 48 h, ZNF750 and RAC1 expressions were determined using (a) Western blotting and (b) quantitation. After transfecting with ZNF750 mimics or inhibitor, the expression levels of ZNF750 were determined using (c) Western blotting and (d) quantitation. *P< 0.05 vs. Res 0 μM control

To confirm the molecular mechanism of resveratrol in suppressing the malignant progression of oral squamous cell carcinoma cells, we further determined the expression levels of ZNF750 and RAC1 in CAL-27 cells after treatment with different concentrations of resveratrol. As shown in Figure 4(a-b), resveratrol significantly upregulated ZNF750 expression and downregulated RAC1 level in a concentration-dependent manner, suggesting that resveratrol inhibits the malignant progression of oral squamous cell carcinoma cells via activating ZNF750/RAC1 signaling pathway.

In addition, our experiments above suggested that resveratrol upregulated the tumor suppressor ZNF750 in OSCC cells, leading us to ask whether this activation might help explain the compound’s ability to inhibit the malignant phenotype in these cells. Thus, we transfected CAL-27 cells with plasmids expressing a ZNF750 mimics or inhibitor to over- or under-express ZNF750. As shown in Figure 4(c-d), the expression level was significantly upregulated in cancer cells transfected with ZNF750 mimics, whereas the expression level was dramatically reduced in OSCC cells transfected with ZNF750 inhibitor.

### Regulation of RAC1 expression and OSCC cell growth by resveratrol via ZNF750 induction

**Figure 5.**
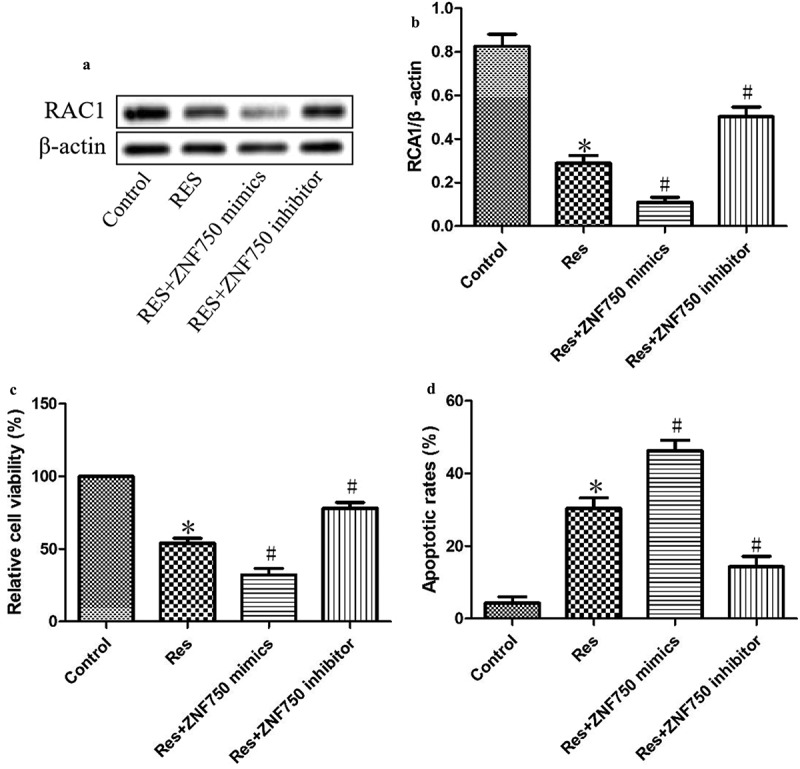
ZNF750 over- or underexpression altered the inhibitory effect of resveratrol (Res) on the growth of OSCC cells. After transfection with ZNF750 mimic or inhibitor for 4 h, CAL-27 cells were treated with 20 μM resveratrol for 48 h, RAC1 expression was determined using (a) Western blotting and (b) quantitation. (c) Cell viability and (d) cell apoptosis were measured using CCK-8 assay and Annexin V-FITC staining, respectively. *P< 0.05, compared to control mimic; ^#^*P*< 0.05 compared to the resveratrol group

As shown in Figure 5(a-b), expression of the mimic resulted in dramatically lower level of RAC1 than expression of the empty vector, whereas expression of the inhibitor led to greatly higher expression of RAC1 protein. Additionally, the expression of the mimic led to significantly lower viability and greater apoptosis than the expression of the empty vector, while the expression of the inhibitor led to significantly higher viability and less apoptosis (Figure 5(c-d)). These results suggest that resveratrol inhibits the growth of OSCC cells by activating the ZNF750 pathway.

### Inhibitory effects of resveratrol on the growth of CAL-27 cells via suppressing RAC1 expression

**Figure 6.**
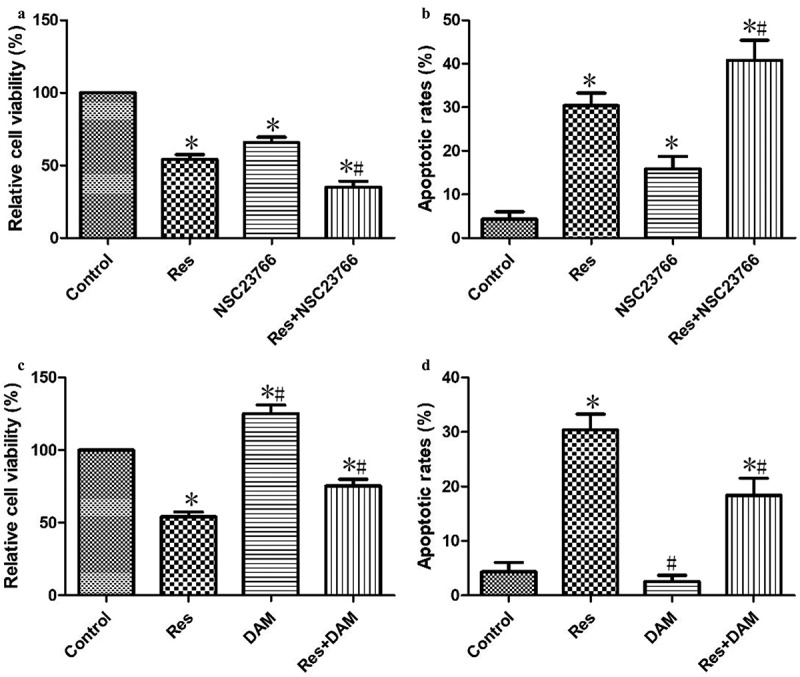
Resveratrol inhibited the growth of CAL-27 cells via suppressing RAC1 expression. After treatment with NSC23766 (2 μM) or DAM (2 μM) for 4 h, and then incubation with 20 μΜ resveratrol for 48 h, the cell viability (a and c) and apoptosis (b and d) were measured using CCK-8 assay and Annexin V-FITC staining, respectively. *P< 0.05, compared to control mimic; ^#^*P*< 0.05 compared to the resveratrol group

In order to verify that the investigated effects of resveratrol on the growth of CAL-27 cells were associated with RAC1 inhibition, cancer cells were treated with RAC1 inhibitor (NSC23766) and agonist (DAM) for 4 h and then incubated with 20 μΜ resveratrol for 48 h. Cells treated with resveratrol and NSC23766 showed significantly lower cell viability (Figure 6(a)), but significantly higher apoptosis (Figure 6(b)) than cells treated only with resveratrol. In addition, cells treated with resveratrol and DAM showed significantly higher cell viability (Figure 6(c)), but significantly lower apoptosis (Figure 6(d)) than cells treated only with resveratrol. These results confirm that resveratrol inhibits CAL-27 cell growth by down-regulating RAC1 expression.

## Discussion

Drugs derived from medicinal herbs show various potent biological functions against multiple cancers [23–27]. Resveratrol is derived from certain plants and shows anti-inflammatory, anti-oxidative stress, and antitumor properties [28–30]. It can modify the tumor microenviroment and sensitize cancer cells to chemo- and radiotherapy [29]. It can also inhibit proliferation and induce apoptosis in several tumor cell types [31,32]. This study extends these activities of resveratrol to OSCC cells, and it suggests that the antitumor efficacy against this disease involves activation of ZNF750-dependent tumor-suppressive pathways.

Previous work has demonstrated that ZNF750 can suppress the malignant progression of OSCCs by regulating the tumor vascular microenvironment [17]. We confirmed and extended those findings by showing that, in OSCC cells, resveratrol downregulates angiogenin, VEGF, RGS5, CD105, and the cell adhesion molecules ITGA5, ITGB1, and CD44 [32]. At the same time, it upregulates PHD2 and PDGFB. These results support the idea that resveratrol inhibits malignant progression in OSCC by regulating the tumor vascular microenvironment. Our observation that resveratrol also upregulates ZNF750 suggests that this endogenous tumor suppressor mediates at least some of the changes in the tumor microenvironment. It is well known that suppressed genes have been enriched for terms related to cell proliferation. Earlier researches reported that ZNF750 is typically mutated or deleted in squamous cell carcinoma [6–8]. The loss of ZNF750 is related to impaired differentiation and failure to fully inhibit the proliferative genetic program, both of which are important markers of tumor [33]. Previous study has demonstrated that ZNF750 could suppress the malignant progression of OSCCs by regulating tumor vascular microenvironment [17]. Thus, this study further observes the role of ZNF750 in the inhibitory effect of resveratrol on the proliferation of OSCC CAL-27 cells. Western blot analysis displayed that resveratrol induced the up-regulation of ZNF750 expression. Then OSCC CAL-27 cells were transfected with ZNF750 knockdown partially reversed the effects of resveratrol on OSCC cell growth. This idea is strengthened by our observation that ZNF750 knockdown partially reversed the effects of resveratrol on OSCC cell growth.

Previous studies have demonstrated that RAC1 is involved in the cell migration and survival in oral cancers [34–36]. Our observations have reported that resveratrol inhibited cell proliferation and induced apoptosis in CAL-27 cells by suppressing the expression of RAC1. In addition, ZNF750 overexpression also decreased the RAC1 expression, whereas its underexpression induced RAC1 level, which is consistent with previous investigation [16]. In our study, RAC1 agonist partially reversed the inhibitory effects of resveratrol on OSCC cell growth. Collectively, the ability of resveratrol to suppress the progression of OSCC may involve activation of the ZNF750/RAC1 signaling pathway and modification of the tumor vascular microenvironment.

Our results justify further work into how resveratrol may induce the activation of ZNF750/RAC1 signaling pathway and how this affects downstream signaling and gene expression pathways. This future research may help develop novel therapeutic targets against OSCC and potentially other cancers in which resveratrol shows antitumor efficacy.

## Conclusion

In summary, resveratrol could regulate the tumor vascular microenvironment to suppress the oral squamous cell carcinoma malignant process through the activation of ZNF750/RAC1 signaling pathway. The results of this study provide a reference for clinical treatment of oral squamous cell carcinoma.
